# Clinical and radiographic outcomes of dental implant after maxillary sinus floor augmentation with rhBMP-2/hydroxyapatite compared to deproteinized bovine bone

**DOI:** 10.1371/journal.pone.0273399

**Published:** 2022-08-25

**Authors:** Jeong Joon Han, Ji Eun Moon, Eun-Hyuk Lee, Hoon Joo Yang, Soon Jung Hwang

**Affiliations:** 1 Department of Oral and Maxillofacial Surgery, School of Dentistry, Seoul National University, Seoul, Republic of Korea; 2 Dental Research Institute, Seoul National University, Seoul, Republic of Korea; 3 Department of Oral and Maxillofacial Surgery, Seoul National University Dental Hospital, Seoul, Republic of Korea; 4 Department of Prosthodontics, School of Dentistry, Seoul National University, Seoul, Republic of Korea; 5 Hwang Soon Jung’s Dental Clinic for Oral and Maxillofacial Surgery, Seoul, Republic of Korea; Virginia Commonwealth University, UNITED STATES

## Abstract

**Objectives:**

This study aimed to evaluate the clinical and radiographic outcomes of early implant placement and functional loading in maxillary sinus floor augmentation (MSFA) using recombinant human bone morphogenetic protein 2/hydroxyapatite (rhBMP-2/HA) and to compare these outcomes with those of the conventional protocol in MSFA using deproteinized bovine bone (DBB).

**Materials and methods:**

The rhBMP-2/HA and DBB groups consisted of 14 and 13 patients who underwent MSFA with BMP and DBB, respectively. After placement of 22 implants and 21 implants in the rhBMP-2/HA and DBB groups, respectively, abutment connections were performed 3 months after implant placement for the rhBMP-2/HA group and 6 months after implant placement for the DBB group. Changes in grafted sinus height (GSH), marginal bone loss (MBL), and implant stability were evaluated up to one year after functional loading.

**Results:**

Survival rates for the rhBMP-2/HA and DBB groups after one year of functional loading were 90.9% and 90.5%, respectively. Both groups exhibited no significant time-course changes in GSH until one year of functional loading (rhBMP-2/HA, *p* = 0.124; DBB, *p* = 0.075). Although significant MBL occurred after one year of functional loading for both groups (rhBMP-2/HA, *p* < 0.001; DBB, *p* < 0.001), there were no significant differences in time-course changes in MBL between the two groups (*p* = 0.450). The mean implant stability quotient values in the rhBMP-2/HA and DBB groups were 75.3 and 75.4 after one year of functional loading, respectively, and there were no significant differences between the two groups (*p* = 0.557).

**Conclusions:**

MSFA using rhBMP-2/HA allowed implant rehabilitation with early implant placement and functional loading and led to a comparable survival rate and implant stability after 1 year of functional loading with acceptable MBL and stable maintenance of GSH compared to the MSFA using DBB with 6 months of healing after implant placement.

## Introduction

Rehabilitation of edentulous areas in the posterior maxilla using dental implants has been established as a successful and satisfactory treatment method [1, 2]. However, it is often difficult to place implants in an ideal position functionally and aesthetically while receiving sound bone support due to alveolar bone resorption after tooth loss, severe periodontitis, and pneumatization of the maxillary sinus [3]. Maxillary sinus floor augmentation (MSFA) is a procedure that enables placement of dental implants with an appropriate length by increasing the available bone height through a sufficient amount of bone grafting after elevation of the sinus membrane [4, 5]. MSFA has been used as a well-accepted surgical procedure to overcome insufficient bone height in the posterior maxilla and to achieve long-term stability [6–8].

Various bone grafting materials, such as autogenous bone, allogeneic bone, xenogeneic bone, and synthetic bone substitutes, have been used to fill the subantral space after elevation of the maxillary sinus membrane in MSFA [9–13]. Since autogenous bone can induce the most effective bone regeneration due to its osteogenic, osteoinductive, and osteoconductive properties, it has been regarded as an ideal and standard graft material for MSFA [14]. However, additional surgery is required in other parts of the body to harvest autogenous bone, and this can increase the patients’ postoperative discomfort, recovery period, and risk of complications. In addition, autogenous bone grafts have limitations in that the amount of bone that can be harvested is limited with a high resorption rate after grafting. To overcome these inherent drawbacks of autogenous bone grafts, various bone substitutes have been developed, and their clinical application and effectiveness have been extensively investigated in the literature. However, bone substitutes usually require a long maturation time after grafting to obtain acceptable bone quality for implant placement [10, 15, 16].

Recombinant human bone morphogenetic protein 2 (rhBMP-2) is a member of the transforming growth factor-β superfamily that induces rapid new bone formation by promoting differentiation of mesenchymal stem cells into osteoblasts [17, 18]. Based on the successful results of preclinical studies and clinical trials, rhBMP-2 has been used to improve bone regeneration in the oral and maxillofacial regions [19, 20]. Previous studies have evaluated new bone formation with the use of rhBMP-2 combined with an absorbable collagen sponge (ACS) and reported that the induced bone in the rhBMP-2/ACS group was significantly denser than that in the bone graft group [18, 21–24]. In addition to ACS, particulate bone substitutes have been used as carriers of rhBMP-2 for volume stability and rigidity. Rapid new bone formation was observed after MSFA using rhBMP-2 with hydroxyapatite (rhBMP-2/HA), and bone formation after 3 months of MSFA using rhBMP-2/HA was comparable to bone formation after 6 months of MSFA using deproteinized bovine bone alone (DBB) [23].

Although several previous studies have reported rapid new bone formation with high bone density in MSFA using rhBMP-2, there are few studies conducted to determine whether the new bone formation achieved through the use of rhBMP-2 and bone substitutes for MSFA is appropriate for implant placement and whether the treatment outcome after early implant placement and functional loading based on rapid new bone formation is successful [18, 21–24]. The purpose of this study was to evaluate the clinical and radiographic outcomes after early implant placement and functional loading in patients who underwent MSFA using rhBMP-2/HA. The null hypothesis was that the treatment outcomes after early implant placement and functional loading for MSFA with rhBMP-2/HA would be comparable to the treatment outcomes of conventional functional loading protocols after MSFA with DBB.

## Materials and methods

To address this research purpose, a single-center, open, prospective, non-randomized clinical study was designed and implemented accordingly. The study protocol was approved by the Institutional Review Board of the Seoul National University Dental Hospital (IRB No. CDE10003) and conducted in accordance with the Declaration of Helsinki. Written informed consent was obtained from all the patients after receiving a detailed explanation of the study.

### Sample size calculation

The sample size calculation was based on implant stability quotient (ISQ) values from a previous study [25] and was performed using G*Power 3.1 (Dusseldorf, Germany) with an 80% power and an α value of 0.05 [26]. The sample size calculation suggested that 11 patients in each group were enough to detect a significant difference between the two groups. To compensate for possible dropouts of 10%, a minimum sample size of 13 patients was estimated.

### Patients

Of the patients who underwent MSFA using rhBMP-2/HA or DBB within the previous 3 months, 27 who agreed to participate and met the inclusion/exclusion criteria were included in this study. Inclusion criteria were as follows: 1) age range, 40–70 years; 2) unilateral MSFA with rhBMP-2/HA or DBB through the lateral approach within the previous 3 months; and 3) voluntary participation. The exclusion criteria were as follows: 1) patients with systemic diseases, including uncontrolled diabetes mellitus, hypertension, or hyperparathyroidism; 2) severe cardiovascular, respiratory, digestive endocrine, psychological, or central nervous system diseases; 3) a recent history of myocardial infarction attack; 4) patients receiving antiresorptive drugs such as bisphosphonates; 5) patients with bleeding disorders; 6) a history of malignancy within the past 5 years; 7) alcoholism; 8) at least 10 cigarettes per day; 9) bruxism or clenching; 10) allergy to implant materials; 11) pregnant or breastfeeding women; 12) untreated dental diseases such as periodontitis and stomatitis; 13) no mandibular teeth that occlude with the planned implant; and 14) judged by the investigator to be inappropriate for the trial since it could affect other ethical or clinical results.

Patients were allocated to the rhBMP-2/HA or DBB groups according to the type of graft material used for MSFA, and a detailed explanation of the study protocol to be implemented was provided. The rhBMP-2/HA group consisted of 14 patients who received MSFA with rhBMP-2/HA (Novosis-Dent, CGBio Inc., Gyeonggi-do, Korea). The rhBMP-2/HA was composed of hydroxyapatite granules with a granule size of 0.6 to 1.0 mm and pore size of 200 to 250 μm, lyophilized *Escherichia coli*-derived rhBMP-2, and distilled water. The mixing ratio was 0.5 g of hydroxyapatite granules, 0.5 mg of rhBMP-2, and 0.5 mL of distilled water. The DBB group consisted of 13 patients who underwent MSFA with DBB (Bio-Oss, Geistlich Pharma AG, Wohlhausen, Switzerland) with a granule size of 0.25 to 1.00 mm. Regardless of the patient group, implants of the same diameter (4.0 mm) and length (11.5 mm) were placed.

### Surgical procedures

MSFA was performed under local anesthesia with 2% lidocaine. After performing crestal and vertical releasing incisions followed by raising of a full-thickness mucoperiosteal flap, the lateral surface of the maxilla was exposed. A bony window with a height of 10–15 mm and a width of 15–20 mm was created using a rotary instrument under cooling conditions with physiological saline irrigation. After removal of the bony window, the maxillary sinus membrane was elevated, and graft materials (rhBMP-2/HA or DBB) were placed in the subantral space. The mucoperiosteal flap was repositioned and sutured using resorbable suture materials (Vicryl 4.0, Ethicon Inc., Cincinnati, OH, USA) without application of a barrier membrane at the created window site. Postoperatively, antibiotics (amoxicillin-clavulanate 625 mg three times per day) and analgesics (acetaminophen 650 mg three-times per day) were prescribed to the patient for 7 days.

Three months after MSFA, all the patients underwent implant placement under local anesthesia with 2% lidocaine. A full-thickness mucoperiosteal flap was raised after creating a crestal incision in the patient. Implants with a diameter of 4.0 mm and a length of 11.5 mm (EZ Plus External, Megagen, Gyeongbuk, Korea) were placed according to the manufacturer’s protocol and left submerged. Antibiotics (amoxicillin-clavulanate 625 mg three times daily) and analgesics (acetaminophen 650 mg three times daily) were prescribed for 7 days after implant placement. Regarding the healing period after implant placement, the rhBMP-2/HA group underwent a secondary operation for abutment connections 3 months after implant placement, whereas the DBB group underwent a secondary operation 6 months or more after implant placement according to the conventional protocol. All the patients underwent prosthetic restorations with fixed prostheses at approximately 4–5 weeks after the abutment connection procedure.

### Radiographic examination

#### Grafted sinus height

Grafted sinus height (GSH) was defined as the height from the implant platform to the highest point of the grafted bone. GSH was digitally measured using Image J (National Institutes of Health, Bethesda, MD, USA) for each implant in panoramic radiographs obtained immediately after implant placement, at the abutment connection procedure, and after one year of functional loading. To correct for magnification of the panoramic radiograph, the magnification ratio was obtained for each radiograph by measuring the length of the implant fixture in the panoramic radiograph and converting it to real implant length (11.5 mm in this study), and the ratio was applied for measurement of GSH.

#### Marginal bone loss

To measure peri-implant marginal bone loss (MBL), periapical radiographs were obtained immediately after implant placement, at the abutment connection procedure, and after one year of functional loading using the paralleling technique. For standardization of scanning periapical radiographs, a customized holding device for parallel imaging, such as an XCP, was used for each patient. By adding impression material to the occlusal body of the XCP, a patient-specific bite block was manufactured, and periapical radiographs were obtained at the same location for each patient at every time point. The magnification ratio was calculated by converting the pitch height measured on the periapical radiograph into the known pitch height of the actual implant fixture (0.6 mm), and it was applied for measurement of MBL on the mesial and distal aspects of each implant, and the average value was calculated accordingly.

#### Implant stability

Implant stability was evaluated by resonance frequency analysis using an Osstell Mentor (Osstell, Osstell AB, Gothenburg, Sweden) at the abutment connection and after one year of functional loading and was presented as an ISQ value on a scale from 1 to 100. After a metal transducer was inserted into the implant screw vent, the ISQ was measured twice (in the buccal-lingual and mesial-distal directions) for each implant, and the average value was calculated.

### Statistical analysis

Statistical analysis was performed using SPSS for Windows (Version 25.0; IBM Inc., Chicago, IL, USA). To determine whether the data followed a normal distribution, the Kolmogorov-Smirnov test was performed. Although there was a normal distribution for all continuous variables, a nonparametric test was used to compare the demographic data and ISQ values because of the small sample size. Differences in the demographic data between the two groups were analyzed using a Mann-Whitney U test or Fisher’s exact test. Survival rates between the two groups were compared using Fisher’s exact test. Repeated measures of analysis of variance (RM-ANOVA) were performed to analyze time-course changes in GSH or MBL within each group or between the two groups. A pairwise post hoc test with Bonferroni adjustment was conducted when RM-ANOVA revealed statistically significant changes within each group. For the ISQ value, a Wilcoxon signed rank test was performed to evaluate the changes between the two time points within each group, and the Mann-Whitney U test was conducted for comparisons between the two groups at each time point. Statistical significance was set at *p* < 0.05.

## Results

A total of 43 implants was placed in 27 patients (male:female = 19:8; mean age, 55.9 ± 6.7 years). The rhBMP-2/HA group consisted of 14 patients (male:female = 9:5, mean age, 55.0 ± 7.3 years), and a total of 22 implants was placed in this group (Table 1). The DBB group consisted of 13 patients (male:female = 10:3, mean age, 57.0 ± 6.2 years), and a total of 21 implants was inserted in this group. In the analysis of computed tomography data obtained before MSFA, the residual alveolar bone height at the location where the implant was to be placed was 4.48 ± 2.69 mm in the rhBMP-2/HA group and 3.92 ± 1.71 mm in the DBB group. The healing period from MSFA to implant placement was 2.87 ± 0.13 months for the rhBMP-2/HA group and 2.85 ± 0.12 months for the DBB group. There were no statistically significant differences between the two groups in age, sex distribution, residual alveolar bone height before MSFA, and healing period from MSFA to implant placement (age, *p* = 0.430; sex, *p* = 0.678; residual alveolar bone height before MSFA, *p* = 0.789; healing period after MSFA, *p* = 0.285). The average healing period for each implant from implant placement to abutment connection was 5.26 ± 2.02 months for the rhBMP-2/HA group and 7.22 ± 1.11 months for the DBB group, and the abutment connection was performed at a significantly earlier time point in the rhBMP-2/HA group compared to the DBB group (*p* = 0.005).

**Table 1:** Demographics and clinical characteristics of the patients included in this study.

	rhBMP-2/HA group	DBB group	*p* value
Number of patients	14	13	
Number of implants	22	21	
Age (years)	55.0 ± 7.3	57.0 ± 6.2	0.430*
Sex, n (%)			0.678^†^
Male	9 (64.3)	10 (76.9)	
Female	5 (35.7)	3 (23.1)	
Residual alveolar bone height before MSFA (mm)	4.48 ± 2.69	3.92 ± 1.71	0.789*
Healing period after MSFA (months)	2.87 ± 0.13	2.85 ± 0.12	0.285*
Healing period from implant placement to abutment connection (months)	5.26 ± 2.02	7.22 ± 1.11	0.005*

Age, residual alveolar bone height, healing period after MSFA, and healing period from implant placement to abutment connection were presented as mean ± standard deviation.

rhBMP-2, recombinant human bone morphogenetic protein 2; HA, hydroxyapatite; DBB, deproteinized bovine bone; MSFA, maxillary sinus floor augmentation

*By Mann-Whitney U test

^†^By Fisher’s exact test

### Survival rate

Of the 43 implants included in this study, four failed to osseointegrate, resulting in an overall survival rate of 90.7%. The survival rates of the rhBMP-2/HA and DBB groups after one year of functional loading were 90.9% (20/22) and 90.5% (19/21), respectively. There were no statistically significant differences in survival rate between the two groups (*p* = 0.679). Regardless of the group, all failed implants showed loss of osseointegration before delivery of the final prosthesis after the abutment connection procedure. At the site where the failed implants were removed, implants with a wider diameter were placed 2 to 6 months later and exhibited successful osseointegration.

### Grafted sinus height

Among the 39 implants that survived one year after functional loading, changes in GSH were analyzed for 35 implants in 22 patients (rhBMP-2/HA group– 19 implants in 12 patients; DBB group– 16 implants in 10 patients). One patient in the rhBMP-2/HA group and two patients in the DBB group were excluded from the analysis of GSH due to lack of panoramic radiographs taken after one year of functional loading. Although the rhBMP-2/HA and DBB groups exhibited a reduction in GSH of 0.10 ± 0.98 mm and 0.63 ± 1.42 mm, respectively, during the period from implant placement to one year of functional loading, there were no significant time-course changes in GSH within either group (rhBMP-2/HA group, *p* = 0.124; DBB group, *p* = 0.075) (Table 2). There were no significant differences in the time-course changes in GSH level between the two groups (*p* = 0.265).

**Table 2:** Grafted bone height and marginal bone loss in the radiographic analysis.

		Immediately after implant placement	At abutment connection	After 1 year of functional loading	RM-ANOVA (*p* value)	
					Time	Time × Group
GSH	rhBMP-2/HA	20.96 ± 3.31	20.56 ± 3.36	20.86 ± 3.59	0.124	0.265
	DBB	18.06 ± 2.38	17.49 ± 2.19	17.43 ± 2.26	0.075	
MBL	rhBMP-2/HA	0	0.11 ± 0.28	0.65 ± 0.38	< 0.001	0.450
	DBB	0	0.21 ± 0.50	0.85 ± 0.67	< 0.001	

Data presented as mean ± standard deviation in millimeters.

RM-ANOVA, repeated measures analysis of variance; GSH, grafted sinus height; MBL, marginal bone loss; rhBMP-2, recombinant human bone morphogenetic protein 2; HA, hydroxyapatite.

### Marginal bone loss

**Fig 1:**
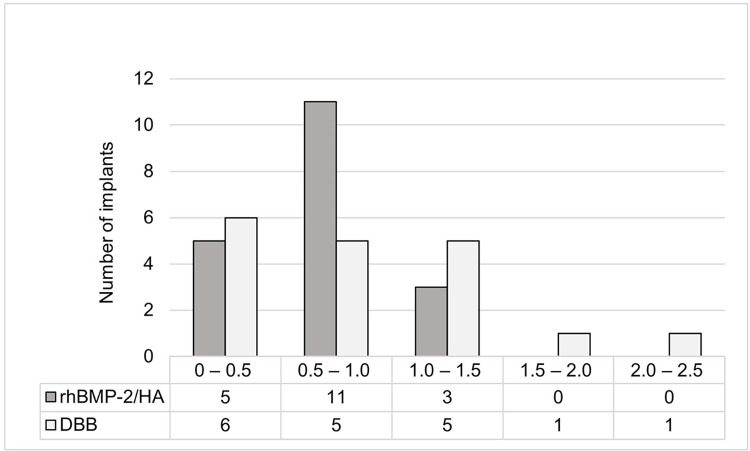
Distribution of marginal bone loss for the rhBMP-2/HA and DBB groups. rhBMP-2, recombinant human bone morphogenetic protein 2; HA, hydroxyapatite; DBB, deproteinized bovine bone.

In the analysis of peri-implant MBL, 37 implants in 23 patients (rhBMP-2/HA group– 19 implants in 12 patients; DBB group– 18 implants in 11 patients) were evaluated accordingly. Of the 39 implants that survived after one year of functional loading, one implant in each group was excluded due to lack of periapical radiographs taken 1 year after functional loading. Both groups exhibited a significant time-course increase in MBL after implant placement to one year of functional loading (rhBMP-2/HA group, *p* < 0.001; DBB group, *p* < 0.001). Compared with the marginal bone level at the time of implant placement, the rhBMP-2/HA and DBB groups exhibited mean MBLs of 0.65 ± 0.38 mm (*p* < 0.001) and 0.85 ± 0.67 mm (*p* < 0.001), respectively, after one year of functional loading (Fig 1). Changes in marginal bone level that occurred during the period from implant placement to the abutment connection procedure were not significant for either group (rhBMP-2/HA group– 0.11 ± 0.28 mm, *p* = 0.311; DBB group– 0.21 ± 0.50 mm, *p* = 0.254). However, significant MBL occurred during the period from abutment connection to one year of functional loading (rhBMP-2/HA group– 0.54 ± 0.36 mm, *p* < 0.001; DBB group– 0.64 ± 0.65 mm, *p* = 0.002). In comparison between the two groups, there were no significant differences in the time-course changes in marginal bone level (*p* = 0.450).

### Implant stability

**Fig 2:**
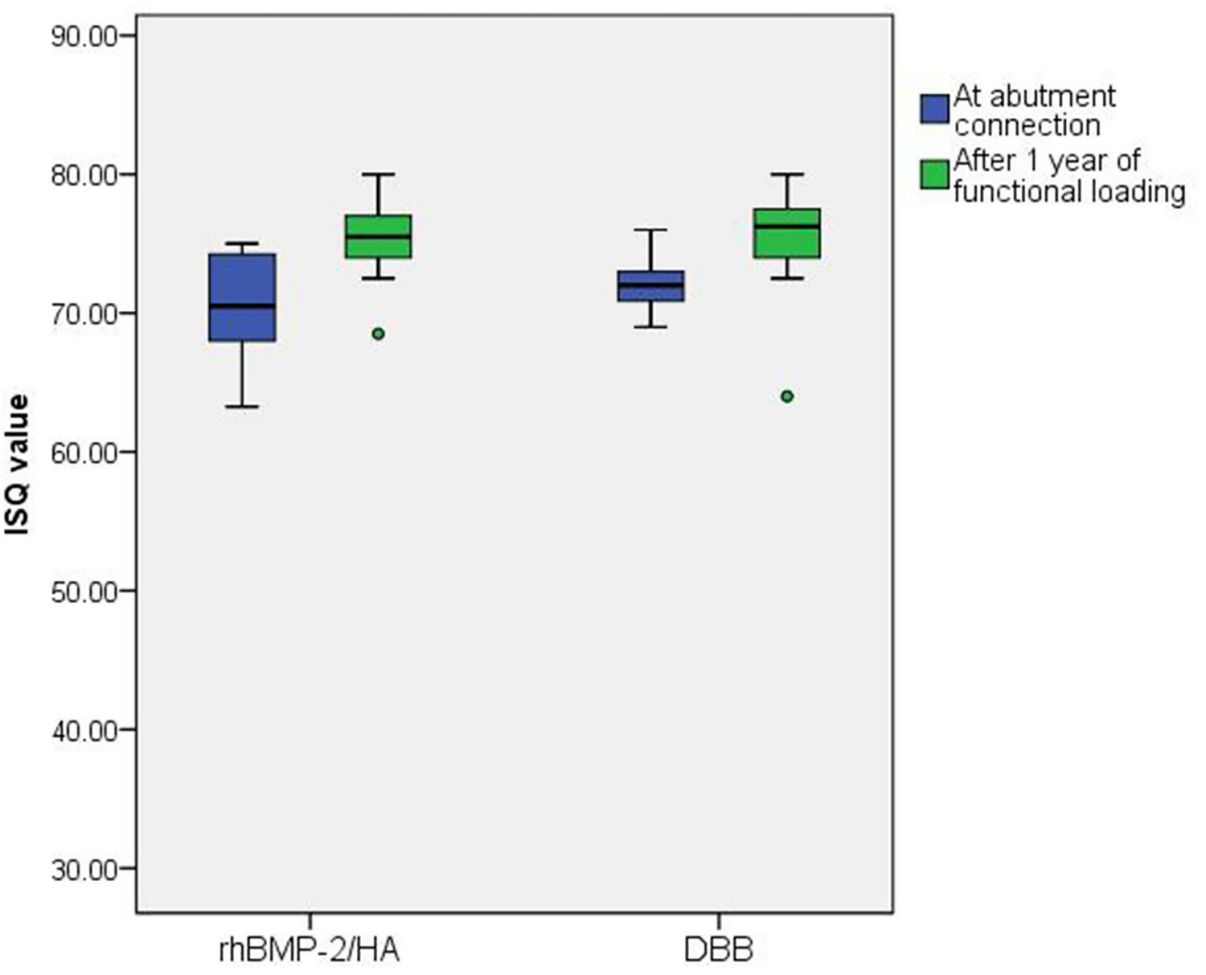
Boxplots presenting ISQ values at abutment connection and after 1 year of functional loading for the rhBMP-2/HA and DBB groups. ISQ, implant stability quotient; rhBMP-2, recombinant human morphogenetic protein 2; HA, hydroxyapatite; DBB, deproteinized bovine bone.

Among the 39 implants that survived one year after functional loading, implant stability was analyzed for 33 implants in 22 patients (rhBMP-2/HA group– 17 implants in 11 patients; DBB group– 16 implants in 11 patients), and three implants in two patients in each group were excluded due to patient rejection of ISQ measurements. The mean ISQ value in the rhBMP-2/HA group was 70.5 ± 3.4 (range of 63.3–75.0) at the time of abutment connection and was 75.3 ± 2.6 (range of 68.5–80.0) after one year of functional loading (Table 3 and Fig 2). The rhBMP-2/HA group exhibited a statistically significant increase in ISQ value (4.8 ± 3.7, *p* = 0.001). In the DBB group, the mean ISQ at the time of abutment connection was 72.0 ± 1.8 (range of 69.0–76.0) with a statistical increase to 75.4 ± 3.7 (64.0–80.0) (increase in ISQ value– 3.4 ± 4.0, *p* = 0.008) after one year of functional loading. In comparison between the two groups, there were no significant differences in ISQ values at the time of abutment connection (*p* = 0.204) and after one year of functional loading (*p* = 0.557). Regarding the mean increase in ISQ value, there were no statistically significant differences between the two groups (*p* = 0.444).

**Table 3:** Changes in ISQ value.

	At abutment connection	After 1 year of functional loading	Changes from abutment connection to 1 year of functional loading
rhBMP-2/HA	70.5 ± 3.4	75.3 ± 2.6	4.8 ± 3.7*
DBB	72.0 ± 1.8	75.4 ± 3.7	3.4 ± 4.0*
*p* value^†^	0.204	0.557	0.444

Data presented as mean ± standard deviation in millimeters.

ISQ, implant stability quotient; rhBMP-2, recombinant human bone morphogenetic protein 2; DBB, deproteinized bovine bone

*p < 0.01 by Wilcoxon signed rank test

^†^By Mann-Whitney U test

## Discussion

In the present study, the clinical and radiographic outcomes of dental implants placed in the posterior maxilla after MSFA using rhBMP-2/HA were investigated and compared with those of dental implants placed after MSFA using DBB. While a similar healing period from MSFA to implant placement was allowed for both groups, the rhBMP-2/HA group received abutment connections and applications of functional loading significantly earlier than the DBB group. After one year of functional loading, the rhBMP-2/HA group exhibited comparable survival rate, implant stability, and changes in GSH and MBL compared to the DBB group despite a significantly shorter healing period from implant placement to abutment connection and functional loading.

Dental implant placement is usually performed 6 months after MSFA to allow maturation of the grafted material, and transmucosal abutment connections and applications of functional loading are performed after an additional healing period of 4–8 months after implant placement [15, 27–30]. To improve bone regeneration and shorten the long treatment timeline from MSFA to completion of prosthetic rehabilitation, the effects of various bioactive proteins have been investigated [22, 31–34]. Platelet-rich fibrin (PRF), an autologous source of platelet derived growth factor, TGF-β, and VEGF, allowed early implant placement and accelerated bone healing with stable maintenance of graft volume [35, 36]. The newly formed bone at 4 months after MSFA using DBB with leukocytes and PRF was significantly greater than at 8 months after MSFA using DBB only [36]. Similarly, with the use of advanced PRF, no significant differences were observed in bone healing at the implant site and in implant stability between the healing periods of 3 months after MSFA and 6 months after MSFA [37]. In addition, heterologous bioactive proteins, such as recombinant human platelet-derived growth factor and rhBMPs, were also evaluated for potential in enhanced bone healing after MSFA [31, 33]. In the study evaluating the effect of rhPDGF, bone formation after MSFA with rhPDGF and DBB was greater than that with DBB only [33]. With respect to rhBMP-2, which is one of the most actively investigated growth factors, rhBMP-2/ACS induced adequate bone formation for placement and functional loading of dental implants in MSFA and exhibited greater bone density than autogenous bone grafting [18, 22]. In the MSFA using rhBMP-2 and DBB, new bone formation at 3 months after MSFA was comparable to that at 6 months after MSFA using DBB only, suggesting the possibility of shortening the treatment period through early implant placement [11, 23, 38, 39]. The enhancing effect of rhBMP-2 on bone formation has been reported to be similar to that of PRF. In the present study, based on the previous histological results, early implant placement and early functional loading were performed after MSFA using rhBMP-2/HA. Despite the significantly shorter healing period after implant placement in rhBMP-2, a clinically acceptable survival rate comparable to that in previous studies was achieved [10, 40, 41].

In the present study, of the 43 total implants, four failed, which might be related to the residual alveolar bone height before MSFA. Regarding the factors affecting the survival rate of implants placed after MSFA, previous studies have reported different conclusions on various possible risk factors, such as graft material, smoking, age, surgical technique, and implant proximity. However, residual alveolar bone height has been suggested as the major risk factor for implant failure in several studies [7, 42–44]. Residual alveolar bone height less than 3 or 4 mm has been significantly associated with implant failure [7, 43, 44]. In a retrospective multicenter study, of the possible risk factors for implant failure in MSFA, residual alveolar bone height less than 4.0 mm and smoking habits exhibited significant association with reduced implant survival [43]. Similarly, in a recent retrospective study, the cumulative survival rate of implants placed in residual bone height less than 3.0 mm (78.8%) was significantly lower than that in residual bone height equal to or greater than 3.0 mm (92.4%) [7]. Consistent with the results in previous studies, all failed implants in the present study exhibited severe alveolar bone insufficiency less than 3.0 mm before MSFA, and the survival rate of implants placed in residual bone height less than 3.0 mm was 75%. Two failed implants in the rhBMP-2/HA group had preoperative residual alveolar height of 2.8 mm and 2.9 mm, and two failed implants in the DBB group had 1.0 mm and 1.6 mm of preoperative residual alveolar bone height. On the other hand, implants placed in residual alveolar bone height equal to or greater than 3.0 mm exhibited a 100% survival rate. Considering that 3 months were provided as the maturation period of grafted bone in both groups, which was significantly shorter than the usual healing period, it might be necessary to consider a longer healing period in patients with severe residual alveolar bone insufficiency, even when rhBMP-2 is used in MSFA.

Volume stability of the grafted bone in the augmented maxillary sinus is important for long-term success of implants and is dependent on the characteristics of the grafted bone materials [30, 45]. Since autogenous bone grafting, which is regarded as the gold standard for restoration of bone defects, undergoes progressive resorption after MSFA with high morbidity of the donor site and increased operation time, DBB or slowly resorbing synthetic bone substitute is usually used alone or mixed with other fast resorbing bone substitute for MSFA [28, 46, 47]. In the up-to-4-year clinical study, DBB exhibited resorption of 0.6 mm on average with a range of 0 to 1.5 mm when used for MSFA [8]. During a 5-year timeframe after MSFA using DBB, 50% of patients exhibited no reduction in GSH, and only 4.2% of patients showed a reduction of up to 50% [30]. In the retrospective radiographic study by Galindo-Moreno, Fernandez-Jimenez [28], the grafted mixture of DBB and autogenous bone exhibited resorption in GSH of 1.05 mm and 1.95 mm in the external connection type and of 1.35 mm and 2.26 mm in the internal connection type at 6 months and 18 months after prosthetic loading, respectively. Hydroxyapatite, which was used as a carrier for rhBMP-2 in this study, is also a slowly resorbing bone substitute. As expected, both the rhBMP-2/HA and DBB groups exhibited stable maintenance of GSH from implant placement to one year of functional loading with a clinically not significant reduction. Although the DBB group exhibited a relatively large decrease in GSH compared to the rhBMP-2/HA group, it was similar to or less than the results of previous studies [8, 28, 30, 48].

MBL is another important factor determining the success and longevity of dental implants [49, 50]. Most early instances of MBL occur within one year after functional loading [51–53]. Several investigators have suggested reference values of MBL from 1.0 to 2.0 mm for successful implants during the first year of functional loading [54–56]. Several studies have suggested that the characteristics of the grafted material could influence the load distribution and MBL around implants placed in the grafted maxillary sinus [57, 58]. When the grafted bone exhibits lower stiffness than the native bone, increased stress at the crestal level can occur under functional loading, leading to MBL [59]. Kim, Yun [48] evaluated MBL around implants placed in grafted maxillary sinuses using DBB and a minimal amount of autogenous bone and reported an average MBL of 0.63 ± 0.51 mm and 0.73 ± 0.52 mm at one year and 20.8 months after functional loading, respectively. They also reported that three of the 49 implants placed exhibited bone resorption greater than 1.5 mm within one year of functional loading. In the study by Galindo-Moreno, Fernandez-Jimenez [28] where the patients received MSFA using a mixture of autogenous bone and DBB at a 1:1 ratio, implants with external connections exhibited MBL of 1.14 mm on the mesial aspect and 1.37 mm on the distal aspect 6 months after functional loading and of 1.93 mm on the mesial aspect and 2.16 mm on the distal aspect 18 months after functional loading. In the present study, the DBB group exhibited a mean MBL of 0.85 mm after one year of functional loading, which is consistent with the results of previous studies. The rhBMP-2/HA group also exhibited a similar mean MBL of 0.65 mm, with most of the MBL occurring after abutment connection procedure in both groups. Maximum MBL in the rhBMP-2/HA and DBB groups was 1.27 mm and 2.3 mm, respectively. In terms of the implant-abutment connection, which is regarded as one of the influencing factors for marginal bone loss, all implants placed in this study had an external implant abutment connection and exhibited similar MBL to the results of a recent systematic review in which MBL in external connections ranged from 0.16 mm to 1.63 mm [60].

In MSFA, the ISQ value can be influenced by the stiffness of the implant-bone interface, geometry of the implant, and bone regeneration surrounding the implant [12, 27, 61]. In the present study, there was no significant difference in residual alveolar bone height before MSFA between the rhBMP-2/HA and DBB groups, and all implants placed in both groups were of the same length and diameter. Therefore, bone regeneration around the implant, such as the amount of surrounding bone and its quality, can be considered as a contributing factor that can affect the difference in ISQ values between the two groups. However, although the rhBMP-2/HA group had a significantly shorter healing period after implant placement compared to the DBB group, implant stability after one year of functional loading for the rhBMP-2/HA group was comparable to the DBB group. Considering that an ISQ value of 60 to 70 indicates sufficient implant stability [61–63], both groups demonstrated high implant stability at the time of abutment connection and after one year of functional loading.

Previously, various graft materials have been used as a carrier for rhBMP-2 in MSFA, such as ACS, DBB, beta-tricalcium phosphate (β-TCP), and HA [12, 23, 64, 65]. ACS exhibits ease of handling and good biocompatibility. However, its lack of mechanical stability and rapid resorption are inadequate for maintaining the grafted bone volume in the maxillary sinus [64]. DBB has a potential risk for transmission of infectious diseases [65]. Also, the addition of rhBMP/ACS to DBB resulted in a negative effect on new bone formation after MSFA compared to DBB alone [66]. For β-TCP, although good biocompatibility and osteoconductivity have been reported, the stability of the augmented grafts was insufficient, and new bone formation was no better that the conventional treatment using DBB [12, 40]. To mitigate the weakness in the physical properties of β-TCP, it is used in the form of biphasic tricalcium phosphate, which is composed of β-TCP and HA. HA, which was used in this study, is a low-biodegradable synthetic bone substitute with a high affinity for rhBMP-2 [67, 68]. It is helpful in maintaining space for induced bone and grafted volume through good structural integrity [23]. In previous animal and human studies, successful treatment outcomes with use of HA as a carrier for rhBMP-2 have been reported [23, 69]. Based on these findings, HA was used as a carrier of rhBMP-2 to be grafted in the maxillary sinus in this study.

rhBMP-2 provides the possibility of early implant placement and early loading through enhanced bone regeneration based on its high osteoinductivity. However, there are several considerations when selecting rhBMP-2 as a graft material compared to DBB. First, rhBMP-2 is expensive, and the patient’s financial burden for the material increases approximately 1.5–2 times compared to using DBB alone. Further, since an additional preparation process of mixing BMP-2, HA, and distilled water is required for grafting BMP-2/HA, the operation time can be increased or an additional assistant might be required. On the other hand, DBB is simple and easy to use because it does not require additional assistants or any further preparation other than hydration of the graft material.

This study has several limitations. The major drawback of the study is the lack of histological evaluation through core bone biopsies during implant site preparation. Therefore, it was difficult to investigate the characteristics of the bone at the site of implant placement as well as the amounts of connective tissue, remaining grafted particles, and newly formed bone. Another limitation is that changes in grafted bone and marginal bone level were measured using two-dimensional analysis on panoramic and periapical radiographs. A three-dimensional analysis using cone-beam computed tomography would have provided a more accurate evaluation of the morphological changes of the peri-implant alveolar bone and volumetric changes of the grafted bone. Regarding primary implant stability, although the stability of the implant immediately after implant placement was clinically confirmed, accurate measurement of implant insertion torque value would have been helpful for strengthening the study conclusion. Compared with several previous studies that only included patients with residual alveolar bone height less than 4 mm, the mean residual alveolar bone height in this study was 4.48 mm in rhBMP-2/HA and 3.92 mm in DBB, which are relatively large. The study would be more relevant if it was conducted only on patients with severe alveolar bone deficiency of 3–4 mm or less. Although the rhBMP-2/HA group exhibited a significantly shorter healing period after implant placement compared to the DBB group, the healing period of the rhBMP-2/HA group was longer than the planned 3 months due to patient circumstances. Last, among the patients who had already undergone maxillary sinus floor augmentation with rhBMP-2/HA or DBB within 3 months before the study, patients who agreed to participate in the study and met the inclusion/exclusion criteria were included. Therefore, it was difficult to randomize patients into the test and control groups at the beginning of the study. As a result, to draw definitive conclusions, a randomized, well-controlled prospective long-term clinical study with three-dimensional computed tomography analysis in a large number of patients will be necessary in the future.

## Conclusion

MSFA using rhBMP-2/HA allowed implant rehabilitation with early implant placement and functional loading and led to a comparable survival rate with implant stability after 1 year of functional loading with acceptable MBL and stable maintenance of GSH compared to MSFA using DBB with 6 months of healing after implant placement.
